# The cost-effectiveness of cemiplimab plus chemotherapy as the first-line treatment for advanced non-small cell lung cancer

**DOI:** 10.3389/fphar.2023.1171302

**Published:** 2023-07-26

**Authors:** Tingting Lu, Yufan Huang, Zhongjie Cai, Wangchun Lin, Xiaoxiao Chen, Ruijia Chen, Yingying Hu

**Affiliations:** ^1^ Department of Pharmacy, Mindong Hospital Affiliated to Fujian Medical University, Ningde, Fujian, China; ^2^ Department of Pharmacy, Mengchao Hepatobiliary Hospital of Fujian Medical University, Fuzhou, Fujian, China

**Keywords:** cost-effectiveness, cemiplimab, chemotherapy, advanced non-small cell lung cancer, first-line treatment

## Abstract

**Background:** The EMPOWER-LUNG 3 clinical trial has shown that cemiplimab plus chemotherapy (CCT) significantly extended overall survival (OS) and progression-free survival (PFS) for patients with advanced non-small cell cancer (NSCLC) compared to placebo plus chemotherapy (PCT). However, the cost-effectiveness of this new treatment option remains unknown. Thus, we evaluated the cost-effectiveness of CCT versus (vs.) PCT as the first-line treatment for patients with advanced NSCLC from the perspective of the Chinese healthcare system.

**Methods:** We constructed a Markov model to evaluate the cost-effectiveness of CCT as the first-line treatment for patients with advanced NSCLC. The transition probabilities were extracted from the survival data of the EMPOWER-LUNG 3 trial. The drugs’ costs were referred from national tender prices, while other model input parameters were derived from the EMPOWER-LUNG 3 trial and published literature. The outcome parameters mainly included quality-adjusted life years (QALYs) and incremental cost-effectiveness ratios (ICERs). One-way sensitivity analysis and probabilistic sensitivity analysis were performed to evaluate the robustness of the model outcomes.

**Results:** Compared to PCT, in the CCT regimen, an additional $79,667 was spent in terms of the total cost and with an additional 0.31 QALYs, resulting in an ICER value of $253,148/QALY. Sensitivity analysis indicated that the hazard ratio (HR) of OS, the cost of cemiplimab (100 mg), and the HR of PFS, all significantly impacted the model’s results. The probability of CCT (vs. PCT) being cost-effective was 0% at a willingness-to-pay threshold of $38,201/QALYs in China. The scenario analysis showed that when the price of cemiplimab was reduced to less than $184.09/100 mg, the CCT regimen could be considered cost-effective as the first-line treatment for patients with advanced NSCLC compared to the PCT.

**Conclusion:** In China, the CCT was not cost-effective as the first-line treatment for patients with advanced NSCLC.

## 1 Introduction

Lung cancer is a common public health concern with the second highest incidence rate among all cancers worldwide and is the leading cause of cancer-related deaths (Sung et al., 2021). Non-small cell lung cancer (NSCLC) accounts for approximately 80%–85% of the common subtypes of lung cancer (Chen et al., 2022b). Unfortunately, the majority of cases (nearly 85%) are already at an advanced stage at the time of first diagnosis (Miller et al., 2022). The overall 5-year survival rate has been less than 5% in the past decade (Arbour and Riely, 2019). Conventional platinum-based chemotherapy is the standard first-line treatment for patients with advanced NSCLC without epidermal growth factor receptor (EGFR) mutations, anaplastic lymphoma kinase (ALK) translocations, or ROS proto-oncogene 1 (ROS1) fusions (Lee, 2019; Ettinger et al., 2022; Leone et al., 2023). However, the clinical benefit of standard chemotherapy remains unsatisfactory, with a 5-year survival rate of only 15% (Rocco et al., 2019; Ibodeng et al., 2023; Lahiri et al., 2023). Therefore, searching for new treatment options is necessary.

In recent years, immune checkpoint inhibitors (ICIs) targeting programmed cell death protein 1 (PD-1) and programmed cell death ligand 1 (PD-L1) have attracted global attention, and their development and application have fundamentally changed the landscape of treatment for newly diagnosed patients with advanced NSCLC, especially those without targeted genetic mutations (Peters et al., 2019). By 2018, approximately 33% of patients diagnosed with advanced NSCLC had received this emerging therapy (American College of Surgeons Commission on Cancer, 2019).

Cemiplimab is an anti-PD-1 monoclonal antibody (Lee et al., 2020). Gogishvili et al. (2022) recently conducted a multicenter phase III clinical trial (EMPOWER-Lung 3; identifier: NCT03409614) and compared the efficacy and safety of cemiplimab plus chemotherapy (CCT) versus (vs.) placebo plus chemotherapy (PCT) as the first-line therapy for untreated patients with locally advanced (stage IIIB or IIIC) or stage IV NSCLC. The outcomes showed that CCT, compared to PCT, had a significant clinical benefit as the first-line treatment for patients with advanced NSCLC, regardless of the levels of PD-L1 expression [median overall survival (OS): 21.9 vs. 13.0 months, hazard ratio (HR) = 0.71; median progression-free survival (PFS): 8.2 vs. 5.0 months, HR = 0.56] (Gogishvili et al., 2022).

Although the CCT regimen, compared to the PCT regimen, showed clear superiority in clinical efficacy as the treatment of advanced NSCLC (Gogishvili et al., 2022), its cost also increased dramatically, which prompted us to consider the following question: is the cost of CCT proportional to its clinical value? Therefore, we evaluated the cost-effectiveness of the CCT regimen as the first-line treatment for patients with advanced NSCLC from the perspective of the Chinese healthcare system in this study. The study aimed to provide healthcare decision makers with economic references for the CCT treatment option and improve the efficient use of limited healthcare resources, especially in cemiplimab pricing and reimbursement. Currently, no other study has evaluated the economics of CCT as the first-line treatment strategy for patients with advanced NSCLC.

## 2 Materials and methods

### 2.1 Model structure

**FIGURE 1:**
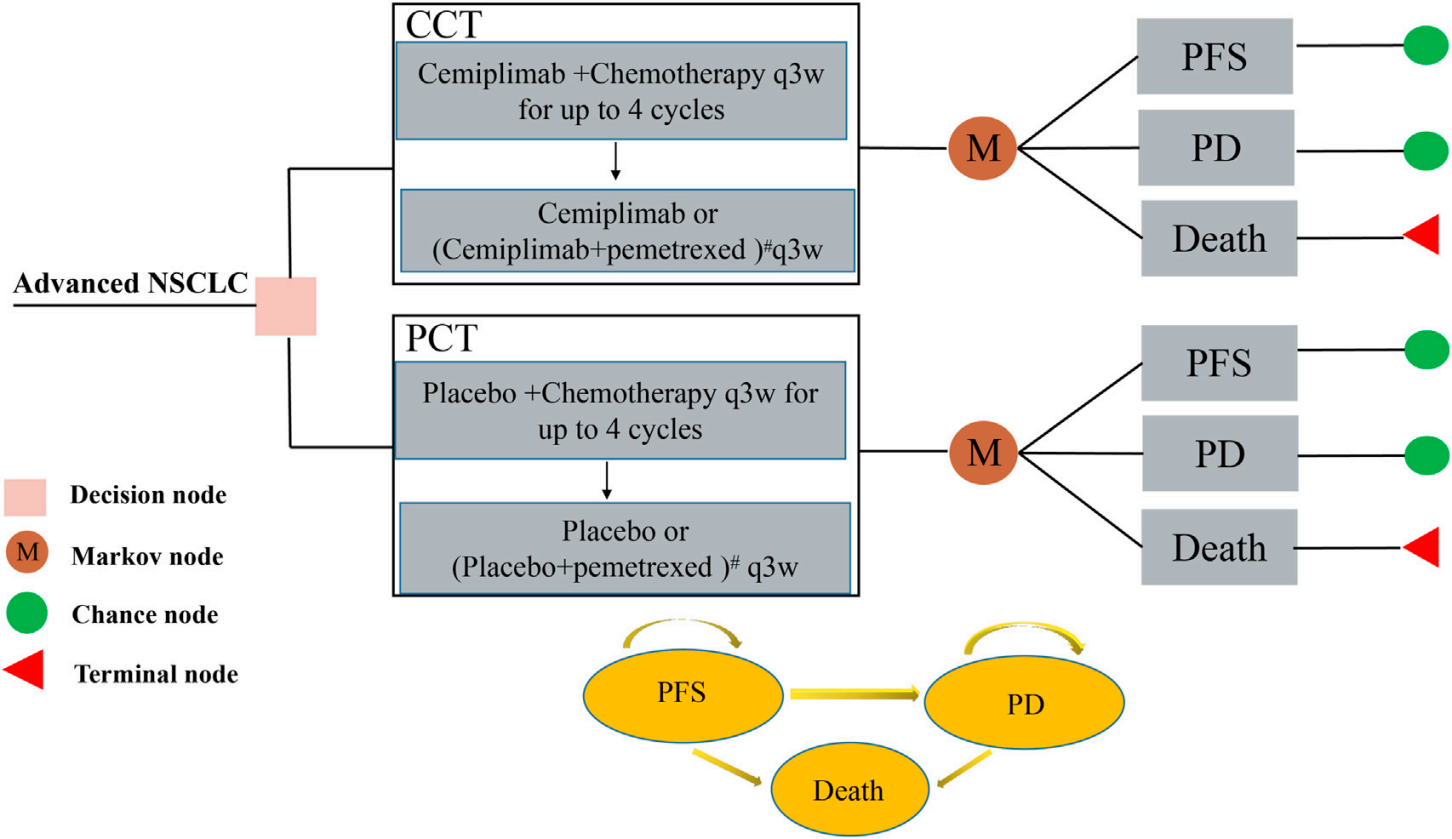
The Markov model used to compare CCT with PCT for treating patients with advanced NSCLC. All patients started with PFS state and randomly received CCT or PCT. # For patients with non-squamous histology who were assigned a pemetrexed-containing regimen, the maintenance regimen was cemiplimab or placebo plus pemetrexed. CCT, cemiplimab plus chemotherapy; NSCLC, non-small cell lung cancer; PCT, placebo plus chemotherapy; PD, progressive disease; PFS, progression-free survival.

The study’s design is based on the consolidated health economic evaluation reporting standards (CHEERS 2022) (Husereau et al., 2022) (Supplementary Table S1). A Markov model was constructed using TreeAge Pro 2022 (TreeAge Software, Williamstown, MA, United States) for evaluating the cost and health outcomes of both CCT and PCT strategies for patients with advanced NSCLC. The model involved three different health states [PFS, progressive disease (PD), and death], according to the progression of advanced NSCLC and relevant references (Liu G. et al., 2021; Luo et al., 2022), as shown in Figure 1. The patient can only be in one of the health states at any given time point. Since both cemiplimab and chemotherapy were administered once every 3 weeks in the EMPOWER-Lung 3 trial, the cycle length of the model was set at 21 days. In each cycle, patients either remained in their previous health state or developed a new one and were not allowed to return to their previous health state. The model’s time horizon was approximately 6 years (determined by assuming the death of 99% of the patients). We assumed that the patients in the model all started with the PFS state and randomly received CCT or PCT.

First, Data points from the Kaplan-Meier survival curves of the PCT group in the EMPOWER-Lung 3 trial were extracted using GetData Graph Digitizer (version 2.26). Pseudo-individual patient data were reconstructed using R software (version 4.2.0) following the algorithm described by Hoyle and Henley (2011). These data were fitted with the following standard parameter models (exponential, Weibull, log-logistic, and log-normal distributions) to obtain survival information over the observation period. Visual inspection and the values of Akaike information criterion (AIC) and Bayesian information criteria (BIC), as indicators of goodness of fit, were compared among the different distribution functions. The lower the values of AIC and BIC, the better the selected model’s fitness (Ishak et al., 2013; Bullement et al., 2019). The Weibull distribution function was found to provide the best fit for the survival data of the PCT group (Supplementary Figure S1). We also estimated the scale parameter (λ) and shape parameter (γ) of the PCT group (Table 1). For the CCT group, λ and γ were calculated according to the following equations: γ _CCT_ = γ _PCT_ and λ _CCT_ = HR × λ _PCT_ (Table 1), where HR is the HR of PFS and OS obtained from the EMPOWER-Lung 3 trial. Finally, the transition probabilities for each cycle in the Markov model were calculated.

**TABLE 1:** Relevant parameters of survival distribution.

Parameters	Value	Source
Weibull survival model of PFS		
CCT	Scale = 0.034608, Shape = 1.388300	Gogishvili et al. (2022)
PCT	Scale = 0.061800, Shape = 1.388300	Gogishvili et al. (2022)
Weibull survival model of OS		
CCT	Scale = 0.021328, Shape = 1.213530	Gogishvili et al. (2022)
PCT	Scale = 0.030040, Shape = 1.213530	Gogishvili et al. (2022)

CCT, cemiplimab plus chemotherapy; OS, overall survival; PCT, placebo plus chemotherapy; PFS, progression-free survival.

### 2.2 Clinical data

The enrolled population, interventions, and survival data for our study were obtained from the EMPOWER-LUNG 3 trial, which enrolled 466 adult patients (≥18 years of age) from 74 medical participating centers of multiple countries with squamous or non-squamous stage IIIB/C (if deemed unsuitable for definitive chemoradiotherapy) or stage IV NSCLC. Patients with tumors positive for EGFR mutations, ALK translocations, or ROS1 fusions were excluded, as well as those who had previously undergone anti-PD-1/PD-L1 therapy (Supplementary Table S2). The patients were randomly assigned in a ratio of 2:1 to the CCT (*n* = 312) or PCT group (*n* = 154) for treatment. Baseline characteristics were generally well-balanced between the two arms. The CCT and PCT arms received cemiplimab and placebo, respectively, 350 mg once every 3 weeks for up to 108 weeks, in combination with a maximum of four cycles of chemotherapy. The chemotherapy regimens were selected based on the patient’s NSCLC histology (Table 2; Supplementary Table S3). After four cycles, pemetrexed (500 mg/m^2^, once every 3 weeks) was maintained for patients with non-squamous histology who were previously assigned to a pemetrexed-containing regimen. Patients were treated until disease progression, unacceptable toxicity, or completion of 108 weeks, whichever occurred first.

**TABLE 2:** Model parameters: baseline values, ranges, and distributions for sensitivity analysis.

Variable	Base value	Range		Distribution	Source
		Min	Max		
Cost($)					
Carboplatin (100 mg)	3.93	3.15	4.72	Gamma	China’s health industry data platform (2022)
Cisplatin (100 mg)	11.25	9.00	13.50	Gamma	China’s health industry data platform (2022)
Cemiplimab (100 mg)	1521.38	1217.10	1825.65	Gamma	China’s health industry data platform (2022)
Pemetrexed (100 mg)	6.51	5.21	7.81	Gamma	China’s health industry data platform (2022)
Paclitaxel (100 mg)	33.68	26.94	40.42	Gamma	China’s health industry data platform (2022)
Neutropenia	82.32	65.86	98.78	Gamma	Li et al. (2021)
Anemia	496.80	397.44	596.16	Gamma	You et al. (2022a)
Routine follow-up per cycle^a^	92.69	74.15	111.22	Gamma	Liu et al. (2021a)
End-of-life care	2665.11	2132.09	3198.13	Gamma	Liu et al. (2021a)
Best supportive care per cycle	365.51	292.41	438.62	Gamma	Liu et al. (2021a)
CCT: Incidence of TEAEs					
Anemia	0.099	0.079	0.119	Beta	Gogishvili et al. (2022)
Neutropenia	0.058	0.046	0.069	Beta	Gogishvili et al. (2022)
PCT: Incidence of TEAEs					
Anemia	0.065	0.052	0.078	Beta	Gogishvili et al. (2022)
Neutropenia	0.059	0.047	0.071	Beta	Gogishvili et al. (2022)
HR of OS	0.710	0.530	0.93	normal	Gogishvili et al. (2022)
HR of PFS	0.560	0.440	0.7	normal	Gogishvili et al. (2022)
Utility value					
PFS	0.804	0.643	0.965	Beta	Nafees et al. (2017)
PD	0.321	0.257	0.385	Beta	Nafees et al. (2017)
Disutility due to TEAEs					
Neutropenia	0.20	0.16	0.24	Beta	Nafees et al. (2017)
Anemia	0.073	0.058	0.088	Beta	Nafees et al. (2017)
Body surface area (m^2^)	1.72	1.38	2.06	Normal	Qiao et al. (2021)
Creatinine clearance rate (ml/min)	70	52.5	87.5	Gamma	Liu et al. (2021b)
Discount rate (%)	5	0	8	Fixed	Chinese Pharmaceutical Association (2020)
Proportion					
First-line chemotherapy treatment: CCT group					
pemetrexed + carboplatin	0.369	0.295	0.442	Beta	Gogishvili et al. (2022)
pemetrexed + cisplatin	0.083	0.067	0.100	Beta	Gogishvili et al. (2022)
paclitaxel + carboplatin	0.494	0.395	0.592	Beta	Gogishvili et al. (2022)
paclitaxel + cisplatin	0.054	0.044	0.065	Beta	Gogishvili et al. (2022)
First-line treatment chemotherapy: PCT group					
pemetrexed + carboplatin	0.299	0.239	0.358	Beta	Gogishvili et al. (2022)
pemetrexed + cisplatin	0.104	0.083	0.125	Beta	Gogishvili et al. (2022)
paclitaxel + carboplatin	0.532	0.426	0.639	Beta	Gogishvili et al. (2022)
paclitaxel + cisplatin	0.065	0.052	0.078	Beta	Gogishvili et al. (2022)

^a^ The cost of routine follow-up per cycle included the cost of outpatient visits, hospitalization, and laboratory tests. CCT, cemiplimab plus chemotherapy; HR, hazard ratio; OS, overall survival; PCT, placebo plus chemotherapy; PD, progressive disease; PFS, progression-free survival; TEAEs, treatment-emergent adverse events.

Because the EMPOWER-LUNG 3 trial lacked subsequent regimens after patients entered the PD state, we referred to some studies (Wu et al., 2020; Chen et al., 2022a; You et al., 2022b), assuming that patients in both the CCT and PCT groups received the best supportive care (BSC) after their disease progressed until death.

### 2.3 Cost and utility estimates

Only direct medical costs were considered, including those of medications, routine follow-up, BSC, end-of-life care, and management of treatment-emergent adverse events (TEAEs) (grade ≥3 and incidence >5%). The prices of drugs were obtained from the national public data platform (China’s health industry data platform, 2022). However, information on the price of cemiplimab in China could not be obtained as it is still unavailable in the Chinese market. Thus, we referred to the Chinese price of a classical PD-1 inhibitor (pembrolizumab, US$2662.41/100 mg) (China’s health industry data platform, 2022) available in China and assumed the same cost of the two drugs for one cycle. Additional data were obtained from published literature (Table 2), wherein costs from previous years were adjusted to those in 2022 in US dollars using the China Statistics Bureau Medical Price Index (National Bureau of Statistics, 2022). All costs were converted to US dollars using an average exchange rate of 1 USD = 6.73 CNY. We obtained utility values of 0.804 and 0.321 for PFS and PD, respectively, from published articles (Nafees et al., 2017; Liu et al., 2020; Zhu et al., 2021). We also considered the disutility of TEAEs (grade ≥3 and incidence >5%) (Zhu et al., 2021). All cost and utility values were discounted at a rate of 5% per year as recommended by the Chinese Guidelines for Pharmacoeconomic Evaluations (Chinese Pharmaceutical Association, 2020). The details of the model input parameter are shown in Table 2.

The major output parameters of the model were total costs, life years (LYs), quality-adjusted life years (QALYs), and incremental cost-effectiveness ratios (ICERs). The QALY value for each treatment option is the sum of the survival time for each state multiplied by the corresponding utility value. The ICER value is the ratio of the difference in cost and the difference in effectiveness between the two groups, which is compared with the given willingness to pay (WTP) threshold. If the ICER was below the WTP threshold, the CCT treatment was considered more cost-effective vs. PCT; The WTP threshold for QALYs, as recommended by the World Health Organization, is three times the national gross domestic product *per capita* in the current year (herein, 2022) (National Bureau of Statistics, 2022), i.e., $38,201/QALY.

### 2.4 Sensitivity analysis

To investigate the effect of the model parameter’s uncertainty on the results, one-way sensitivity analysis and probabilistic sensitivity analysis (PSA) were performed In one-way sensitivity analysis, the ranges of the relevant parameters were their 95% confidence intervals from the EMPOWER-Lung 3 trials or set to be ± 20% of the baseline values if the former were not available (Zhang et al., 2012). For PSA, appropriate distributions were assigned for different types of parameters (Table 2) and 1,000 iterative Monte Carlo simulations were performed. Moreover, we repeatedly calculated the acceptable probabilities of the cost-effectiveness for the CCT regimen by continuously reducing the price of cemiplimab. Then, an appropriate price of cemiplimab was obtained when the acceptance probability was 50% at a WTP threshold of $38,201. When the acceptable probability was above 50%, CCT was considered cost-effective as the first-line treatment for patients with advanced NSCLC.

### 2.5 Subgroup analysis

Subgroup analysis was performed for group characteristics including age, sex, histological characteristics, and PD-L1 expression (Table 3), among others, to determine whether the corresponding performance was better in terms of cost-effectiveness in specific subgroups. Given the lack of Kaplan-Meier survival curves of the subgroup population in the EMPOWER-Lung 3 trial, based on the method described by Hoyle et al. (2010), we assumed that survival data of all subgroups in the PCT group followed the Weibull distribution, while the survival functions of all subgroups in the CCT group was estimated based on the subgroup-specific HRs (Table 3) from the EMPOWER-Lung 3 trial. It needs to be emphasized that all parameters were assumed to be consistent with those of the entire patient population, except for subgroup-specific HRs. Finally, ICERs and cost-effectiveness probabilities were obtained for each subgroup.

**TABLE 3:** Results of subgroup analyses.

Subgroup	PFS_HR (95% CI)	OS_HR (95% CI)	ICER ($/QALY)
Age group (years)			
<65	0.53 (0.39–0.71)	0.57 (0.40–0.81)	199885
≥65	0.56 (0.39–0.81)	0.88 (0.56–1.37)	322140
Sex			
Male	0.48 (0.37–0.61)	0.55 (0.41–0.74)	186283
Female	0.90 (0.50–1.62)	2.11 (0.89–5.03)	−376641
Race			
White	0.54 (0.43–0.69)	0.67 (0.50–0.89)	233047
Non-white	0.58 (0.28–1.20)	0.79 (0.31–2.02)	293647
Histology			
Squamous	0.56 (0.40–0.79)	0.56 (0.37–0.84)	201825
Non-squamous	0.53 (0.39–0.73)	0.79 (0.54–1.14)	270335
PD-L1 expression level			
<1%	0.76 (0.51–1.15)	1.01 (0.63–1.60)	766758
1%-49%	0.47 (0.33–0.68)	0.52 (0.32–0.83)	177590
≥50%	0.47 (0.31–0.72)	0.61 (0.37–1.02)	199170
ECOG PS			
0	0.20 (0.09–0.43)	0.55 (0.20–1.49)	128741
1	0.60 (0.47–0.76)	0.69 (0.52–0.92)	258399
Region			
Europe	0.55 (0.43–0.70)	0.67 (0.50–0.90)	235853
Asia	0.52 (0.25–1.10)	0.72 (0.27–1.88)	243136
Brain metastasis			
Yes	0.53 (0.22–1.31)	0.42 (0.14–1.26)	159009
No	0.54 (0.43–0.69)	0.68 (0.51–0.90)	236383
Cancer stage at screening			
Locally advanced	0.34 (0.19–0.62)	0.54 (0.25–1.15)	160358
Metastatic	0.59 (0.46–0.75)	0.69 (0.51–0.93)	255203
Smoking history			
Smokers	0.53 (0.42–0.68)	0.61 (0.46–0.82)	211747
Never smokers	0.65 (0.34–1.22)	1.28 (0.53–3.08)	899626

CI, confidence interval; ECOG PS, eastern cooperative oncology group performance status; HR, hazard ratio; ICER, incremental cost-effectiveness ratio; OS, overall survival; PD-L1, programmed cell death ligand-1; PFS, progression-free survival; QALY, quality-adjusted life-year.

## 3 Results

### 3.1 Base-case analysis

For the entire patient population, $79,667 was spent more in the CCT group vs. PCT group with an additional 0.31 QALYs, resulting in an ICER value of $253,148/QALY (Table 4), which was much higher than the WTP threshold ($38,201/QALYs) in China. Thus, the CCT was not cost-effective compared to PCT as the first-line treatment for patients with advanced NSCLC in China.

**TABLE 4:** The base results of the cost-effectiveness analysis.

Treatment	Total cost ($)	Total life years	Total QALYs	Incremental cost ($)	Incremental QALY	ICER ($/QALY)
CCT	90558	2.49	1.14	79667	0.31	253148
PCT	10891	1.83	0.83	—	—	—

ICER, incremental cost-effectiveness ratio; CCT, cemiplimab plus chemotherapy; PCT, placebo plus chemotherapy; QALY, quality-adjusted life year.

### 3.2 Sensitivity analyses

#### 3.2.1 One-way sensitivity analysis

**FIGURE 2:**
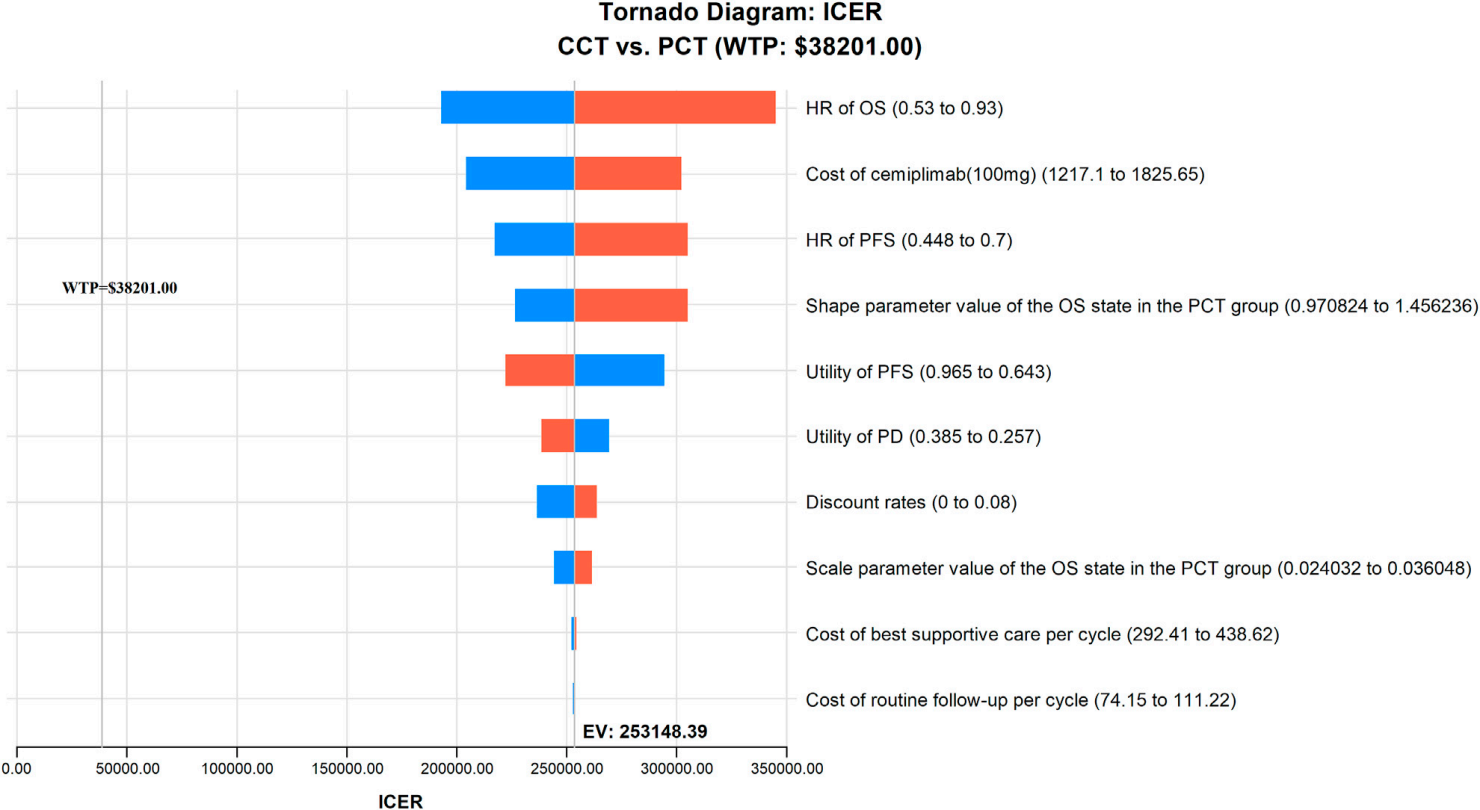
The results of one-way sensitivity analysis indicating the most influential parameters for model’s results in China. CCT, cemiplimab plus chemotherapy; HR, hazard ratio; ICER, incremental cost-effectiveness ratio; OS, overall survival; PCT, placebo plus chemotherapy; PFS, progression-free survival; WTP, willingness-to-pay.

The results of the one-way sensitivity analysis are presented as a tornado diagram (Figure 2). The HR of OS, the cost of cemiplimab (100 mg), the HR of PFS, the shape parameter value of the OS in the PCT group, and the utility values of PFS and PD all had a significant impact on the results of the model. However, the ICER value was always above the WTP threshold regardless of the variation in each parameter within the preset upper and lower limits, thereby confirming that our model outcomes were reliable.

#### 3.2.2 PSA

**FIGURE 3:**
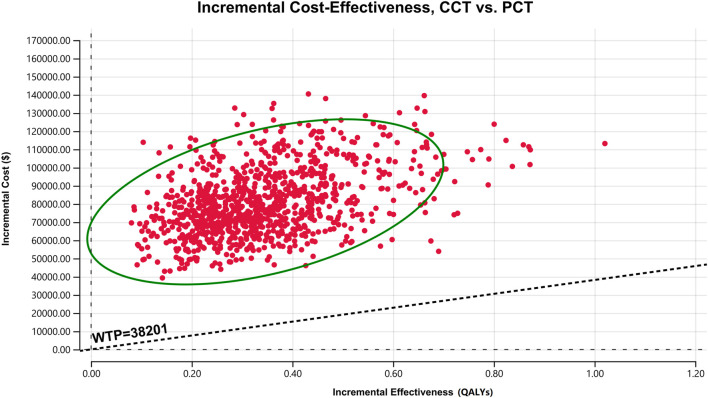
A scatter plot of ICER for probabilistic sensitivity analysis. The points in the graph are obtained by Monte Carlo simulation of 1,000 iterations. Points above the WTP threshold line show the cost-effectiveness of PCT over CCT; conversely, CCT is cost-effective. CCT, cemiplimab plus chemotherapy; ICER, incremental cost-effectiveness ratio; PCT, placebo plus chemotherapy; QALYs, quality-adjusted life years; WTP, willingness-to-pay.

**FIGURE 4:**
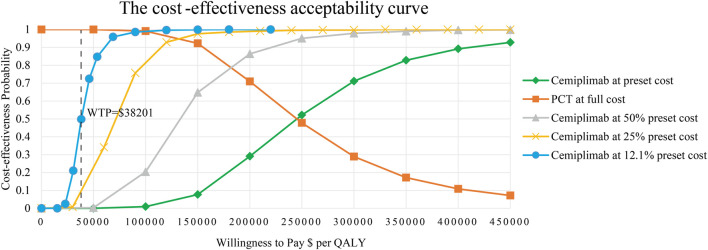
The cost-effectiveness acceptability curves for CCT versus PCT. CCT, cemiplimab plus chemotherapy; PCT, placebo plus chemotherapy; QALY, quality-adjusted life year; WTP, willingness-to-pay.

The results of PSA are presented as a scatter plot (Figure 3) and cost-effectiveness acceptability curves (Figure 4). As shown, the probability that CCT was cost-effective relative to PCT was 0% in China, at a WTP threshold of $38,201. We also found that when the original price of cemiplimab was reduced to 12.10% (i.e., $184.09/100 mg), the probability of CCT being cost-effective rose to 50%, implying that the CCT regimen could be considered cost-effective as the first-line treatment for patients with advanced NSCLC compared to PCT if the price of cemiplimab dropped below $184.09/100 mg.

### 3.3 Subgroup analysis

The results of our subgroup analysis are shown in Table 3. For all subgroups of the population with different characteristics, the ICERs of CCT were all above the WTP threshold of $38,201 with 0% probabilities of being cost-effective compared to PCT.

## 4 Discussion

Indeed, in the era of targeted therapy and immunotherapy, platinum-based compounds and other standard chemotherapeutic agents still represent key treatments for the survival of NSCLC patients (Hellmann et al., 2016; Lee, 2019; Falzone et al., 2023). However, chemotherapy alone has a limited survival benefit and poor prognosis for NSCLC patients (Rocco et al., 2019). According to the recommendations of Guidelines of Chinese Society of Clinical Oncology (CSCO) for NSCLC (2022 edition) (Guidelines Working Committee of Chinese Society of Clinical Oncology, 2022), immunotherapy represented by immune checkpoint inhibitors (e.g., PD-1/PD-L1 inhibitors) has been shown to significantly improve the survival of patients with advanced NSCLC in recent years.

In November 2022, the FDA approved a new indication for cemiplimab based on the results of the EMPOWER-LUNG 3 trial, a phase III clinical trial for cemiplimab, as the first-line treatment of patients with advanced NSCLC without EGFR, ALK, or ROS1 mutations, regardless of the levels of PD-L1 expression. The EMPOWER-LUNG 3 trial showed that the CCT arm demonstrated better clinical benefits compared to the PCT arm, as evidenced by the prolonged OS and PFS of patients with advanced NSCLC However, as in some other countries (Godman et al., 2021), the affordability of innovative anticancer medicines (e.g., PD-1/PD-L1 inhibitors) is a hitherto unknown and grave challenge for the majority of Chinese patients with cancer. One study predicts that the financial burden of cancer in China may continue to rise in the coming decades (Chen et al., 2016). For example, without insurance, patients who were prescribed trastuzumab had to pay more than $50,000 in that year, almost 24 times the Chinese annual *per capita* disposable income during the same period (Chen et al., 2009). The high cost of these innovative anticancer drugs not only places a monetary burden on cancer patients but also exacerbates the drain on healthcare resources, especially for developing countries like China with limited healthcare resources. Therefore, it is essential for us to evaluate the economics of the CCT treatment strategy for these patients compared to PCT from a Chinese perspective.

This is the first study to evaluate the cost-effectiveness of CCT as a first-line treatment option for advanced NSCLC from the Chinese healthcare perspective. Our study included patients with different levels of PD-1 expression and no genetic mutations. The results of this study showed that the ICER of $253,148/QALY in China was above the WTP threshold ($38,201/QALYs), indicating that the CCT treatment strategy was unlikely to provide a proportionate and reasonable value for the money spent. Sensitivity analysis verified that the results were reliable in general. The results of the subgroup analysis showed that the CCT was not a cost-effective choice for the first-line treatment of advanced NSCLC in different subgroups of patients.

According to the recommendations of Guidelines of Chinese Society of Clinical Oncology for NSCLC (2022 edition) (Guidelines Working Committee of Chinese Society of Clinical Oncology, 2022), PD-1/PD-L1 inhibitors in combination with chemotherapy is the major first-line treatment regimen for patients with advanced NSCLC without driver mutations, including pembrolizumab, atezolizumab, camrelizumab, and sugemalimab. Several existing studies (Liu G. et al., 2021) (Wan et al., 2020; Xiang et al., 2021; Zheng et al., 2022) have evaluated the cost-effectiveness of these PD-1/PD-L1 inhibitors in combination with chemotherapy as the first-line treatment for advanced NSCLC from the perspective of Chinese payers. The results of all these studies suggested that the combination of the PD-1/PD-L1 inhibitor and chemotherapy is unlikely to be a cost-effective option for patients with advanced NSCLC compared to chemotherapy alone, consistent with our results. This is due to their modest incremental effect and the high cost of PD-1/PD-L1 inhibitors. However, the previous findings should not be a reason to restrict the use of PD-1/PD-L1 inhibitors, as we risk missing beneficial therapeutic options but rather be treated as economic references for healthcare policymakers in setting a reasonable market price for cemiplimab and in national drug price negotiations. Therefore, we conducted a scenario analysis by continuously reducing the price of cemiplimab to make CCT cost-effective and affordable for patients with advanced NSCLC. The results suggested that the CCT regimen became economical compared to PCT only when the price of cemiplimab was below $184.09/100 mg (probability of cost-effectiveness, >50%).

Some limitations of our study warrant further consideration. First, we extrapolated the survival curves by fitting parameter functions, which inevitably resulted in deviations between the model’s results and the actual situation, an unavoidable drawback of most cost-effective analyses. We will revise our analysis as long-term survival data become available. Second, only a minority of the study population in the EMPOWER-LUNG 3 trial was Chinese (5.58%), which may not accurately reflect the efficacy of Chinese patients. However, we performed a one-way sensitivity analysis of the two key parameters (λ, γ) of the survival curve from the EMPOWER-LUNG 3 trial. The results of the sensitivity analysis suggested that this does not affect the results of our economic evaluation. Third, since cemiplimab is not yet available in China, we used the price of another PD-1 inhibitor, pembrolizumab, which is available in China. We will update the results of our study when the price of cemiplimab is available. Fourth, we assumed that all the patients received BSC after entering the PD state, as EMPOWER-LUNG 3 results for subsequent treatment were not available. Although this assumption may differ from clinical practice, one-way sensitivity analysis suggested that the cost of subsequent treatment was not sensitive to the outcome. When the subsequent treatment plan is released, we will refine the corresponding results. Fifth, due to the lack of corresponding head-to-head trials, we could not directly evaluate the cost-effectiveness of the CCT regimen vs. other PD-1/PD-L1 inhibitors in combination with chemotherapy, recommended by the Guidelines of CSCO for NSCLC (Guidelines Working Committee of Chinese Society of Clinical Oncology, 2022) as potential first-line regimens for patients with advanced NSCLC, like pembrolizumab plus chemotherapy. More clinical trials are required in the future to support this type of cost-effectiveness analysis. Finally, the results of the subgroup analysis should be cautiously interpreted because it is an exploratory study with several unknown parameters. Despite these limitations, we believe that our findings have important economic implications for Chinese decision makers as a reference for drug pricing and health insurance access negotiations.

## 5 Conclusion

In conclusion, for the first time, we conducted an economic evaluation of CCT for patients with advanced NSCLC from the Chinese healthcare perspective. Compared to PCT, CCT was not a cost-effective choice for patients with advanced NSCLC without EGFR mutations or ALK rearrangements in China. A price reduction for cemiplimab may be a potential measure to make CCT cost-effective and affordable after entering China, thus providing more economic options for these patients.

## Data Availability

The original contributions presented in the study are included in the article/Supplementary Material, further inquiries can be directed to the corresponding authors.
