# Supplementary material for: The cost-effectiveness of cemiplimab plus chemotherapy as the first-line treatment for advanced non-small cell lung cancer
Source: Front Pharmacol. 2023 Jul 26;14:1171302. doi: 10.3389/fphar.2023.1171302 (PMC10409993; doi:10.3389/fphar.2023.1171302)
Supplement: Supplementary file 1 [file DataSheet1.docx]

***Supplementary Material***

**The Cost-Effectiveness of Cemiplimab plus Chemotherapy as the First-Line Treatment for** **Advanced Non-Small Cell Lung Cancer**

**Tingting Lu^1†^, Ruijia Chen^2†^, Zhongjie Cai^1^, Wangchun Lin^1^, Xiaoxiao Chen^1^, Yufan Huang^1*^, Yingying Hu^2*^**

^1^Department of Pharmacy, Mindong Hospital Affiliated to Fujian Medical University, Ningde, Fujian, China

^2^Department of Pharmacy, Mengchao Hepatobiliary Hospital of Fujian Medical University, Fuzhou, Fujian, China

*** Correspondence:**

Yufan Huang,

E-mail address: huangyufan2021@126.com

Yingying Hu,

E-mail address: celiahyy@126.com

† These authors have contributed equally to this work

1. Supplementary Table S1. CHEERS 2022 Checklist.

2. Supplementary Figure S1. Comparison of the fitting curves simulated by Weibull distribution functions with the Kaplan-Meier curves from the EMPOWER-LUNG 3 trial for the PCT arm.

3. Supplementary Table S2. The baseline characteristics of patients enrolled in the EMPOWER-LUN G 3 clinical trial.

4. Supplementary Table S3. Choice of chemotherapy options.

1. Supplementary Table S1. CHEERS 2022 Checklist.

| **Topic** | **No.** | **Item** | **Location where item is reported** |
| --- | --- | --- | --- |
| **Title** |  |  |  |
|  | 1 | Identify the study as an economic evaluation and specify the interventions being compared. | Page1 Line1-3 |
| **Abstract** |  |  |  |
|  | 2 | Provide a structured summary that highlights context, key methods, results, and alternative analyses. | Page2 Line37-63 |
| **Introduction** |  |  |  |
| **Background and objectives** | 3 | Give the context for the study, the study question, and its practical relevance for decision making in policy or practice. | Page3 Line81-122 |
| **Methods** |  |  |  |
| **Health economic analysis plan** | 4 | Indicate whether a health economic analysis plan was developed and where available. | Not Applicable |
| **Study population** | 5 | Describe characteristics of the study population (such as age range, demographics, socioeconomic, or clinical characteristics). | Page4 Line162-168 |
| **Setting and location** | 6 | Provide relevant contextual information that may influence findings. | Page4 Line164 |
| **Comparators** | 7 | Describe the interventions or strategies being compared and why chosen. | Page4-5 Line168-170 |
| **Perspective** | 8 | State the perspective(s) adopted by the study and why chosen. | Page5 Line188-191 |
| **Time horizon** | 9 | State the time horizon for the study and why appropriate. | Page4 Line137-138 |
| **Discount rate** | 10 | Report the discount rate(s) and reason chosen. | Page5 Line206-208 |
| **Selection of outcomes** | 11 | Describe what outcomes were used as the measure(s) of benefit(s) and harm(s). | Page5 Line210-211 |
| **Measurement of outcomes** | 12 | Describe how outcomes used to capture benefit(s) and harm(s) were measured. | Page5 Line215-218 |
| **Valuation of outcomes** | 13 | Describe the population and methods used to measure and value outcomes. | Page5-6 Line203-205, 210-213 |
| **Measurement and valuation of resources and costs** | 14 | Describe how costs were valued. | Page5  Line 189-197 |
| **Currency, price date, and conversion** | 15 | Report the dates of the estimated resource quantities and unit costs, plus the currency and year of conversion. | Page5  Line 198-197 |
| **Rationale and description of model** | 16 | If modelling is used, describe in detail and why used. Report if the model is publicly available and where it can be accessed. | Page4  Line 126-139 |
| **Analytics and assumptions** | 17 | Describe any methods for analysing or statistically transforming data, any extrapolation methods, and approaches for validating any model used. | Page4  Line 125-139 |
| **Characterising heterogeneity** | 18 | Describe any methods used for estimating how the results of the study vary for subgroups. | Page6  Line 241-253 |
| **Characterising distributional effects** | 19 | Describe how impacts are distributed across different individuals or adjustments made to reflect priority populations. | Page6  Line 223-230 |
| **Characterising uncertainty** | 20 | Describe methods to characterise any sources of uncertainty in the analysis. | Page6  Line 230-234 |
| **Approach to engagement with patients and others affected by the study** | 21 | Describe any approaches to engage patients or service recipients, the general public, communities, or stakeholders (such as clinicians or payers) in the design of the study. | Not Applicable |
| **Results** |  |  |  |
| **Study parameters** | 22 | Report all analytic inputs (such as values, ranges, references) including uncertainty or distributional assumptions. | Table 2 |
| **Summary of main results** | 23 | Report the mean values for the main categories of costs and outcomes of interest and summarise them in the most appropriate overall measure. | Page6-7  Line 230-234, Table 4 |
| **Effect of uncertainty** | 24 | Describe how uncertainty about analytic judgments, inputs, or projections affect findings. Report the effect of choice of discount rate and time horizon, if applicable. | Page7  Line 261-277 |
| **Effect of engagement with patients and others affected by the study** | 25 | Report on any difference patient/service recipient, general public, community, or stakeholder involvement made to the approach or findings of the study | Not applicable |
| **Discussion** |  |  |  |
| **Study findings, limitations, generalisability, and current knowledge** | 26 | Report key findings, limitations, ethical or equity considerations not captured, and how these could affect patients, policy, or practice. | Page8  Line 323-344 |
| **Other relevant information** |  |  |  |
| **Source of funding** | 27 | Describe how the study was funded and any role of the funder in the identification, design, conduct, and reporting of the analysis | Page9  Line 363-368 |
| **Conflicts of interest** | 28 | Report authors conflicts of interest according to journal or International Committee of Medical Journal Editors requirements. | Page9  Line 369-371 |


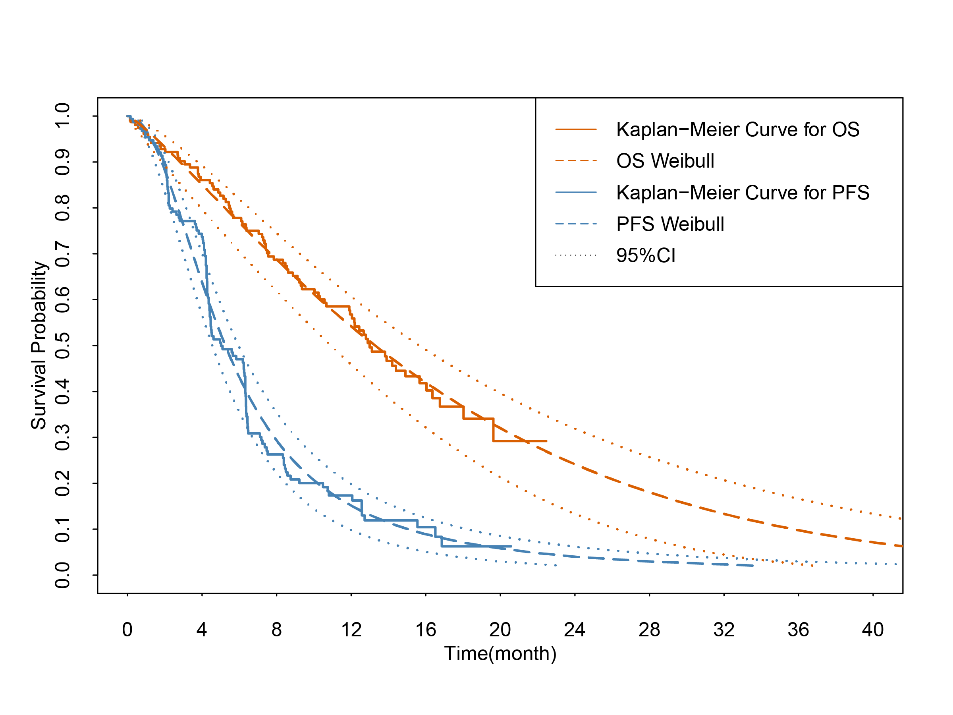
2. Supplementary Figure S1. Comparison of the fitting curves simulated by Weibull distribution functions with the Kaplan-Meier curves from the EMPOWER-LUNG 3 trial for the PCT arm.

CI: confidence interval; OS: overall survival; PCT: placebo plus chemotherapy; PFS: Progression-free survival.

3. Supplementary Table S2. The baseline characteristics of patients enrolled in the EMPOWER-LUN G 3 clinical trial.

| Characteristic | Cemiplimab +   chemotherapy (n= 312) | Placebo +   chemotherapy (n= 154) | Total (n= 466) |
| --- | --- | --- | --- |
| Age, years |  |  |  |
| Median (IQR) | 63.0 (57–68) | 63.0 (57–68) | 63.0(57–68) |
| ≥65, n (%) | 128 (41.0) | 60 (39.0) | 188 (40.3) |
| Sex, n (%) |  |  |  |
| Women | 44 (14.1) | 31 (20.1) | 75 (16.1) |
| Men | 268 (85.9) | 123 (79.9) | 391 (83.9) |
| Geographic region, n (%) |  |  |  |
| Europe | 270 (86.5) | 138 (89.6) | 408 (87.6) |
| Asia | 42 (13.5) | 16 (10.4) | 58 (12.4) |
| Histology, n (%) |  |  |  |
| Non-squamous | 179 (57.4) | 87 (56.5) | 266 (57.1) |
| Squamous | 133 (42.6) | 67 (43.5) | 200 (42.9) |
| PD-L1 expression,n (%) |  |  |  |
| <1% | 95 (30.4) | 44 (28.6) | 139 (29.8) |
| 1–49% | 114 (36.5) | 61 (39.6) | 175 (37.6) |
| ≥50% | 103 (33.0) | 49 (31.8) | 152 (32.6) |
| ECOG PS, n (%) |  |  |  |
| 0 | 51 (16.3) | 18 (11.7) | 69 (14.8) |
| 1 | 259 (83.0) | 134 (87.0) | 393 (84.3) |
| Brain metastasis, n (%) | 24 (7.7) | 7 (4.5) | 31 (6.7) |
| Cancer stage at screening, n (%) | 267 (85.6) | 130 (84.4) | 397 (85.2) |
| Metastatic | 267 (85.6) | 130 (84.4) | 397 (85.2) |
| Locally advanced | 45 (14.4) | 24 (15.6) | 69 (14.8) |
| Smoking history, n (%) |  |  |  |
| Current smoker | 173 (55.4) | 75 (48.7) | 248 (53.2) |
| Past smoker | 96 (30.8) | 55 (35.7) | 151 (32.4) |
| Never smoker | 43 (13.8) | 24 (15.6) | 67 (14.4) |
| Previous cancer-related therapy, n (%) |  |  |  |
| Systemic adjuvant therapy | 5 (1.6) | 1 (0.6) | 6 (1.3) |
| Systemic other | 1 (0.3) | 0 | 1 (0.2) |
| Radiotherapy | 40（12.8） | 11（7.1） | 51（10.9） |

ECOG PS, eastern cooperative oncology group performance status; PD-L1, programmed cell death ligand-1;

4. Supplementary Table S3. Choice of chemotherapy options

| Option | Chemotherapy | Dosing frequency |
| --- | --- | --- |
| 1 | Paclitaxel 200 mg/m^2^ plus carboplatin (AUC of 5 mg/mL/minute), IV | Day 1 every 21 days for 4 cycles; calculate the dose of carboplatin using the Calvert formula |
| 2 | Paclitaxel 200 mg/m^2^ plus cisplatin 75 mg/m^2^, IV | Day 1 every 21 days for 4 cycles |
| 3 | Pemetrexed 500 mg/m^2^ plus carboplatin (AUC of 5 mg/mL/minute), IV | Day 1 every 21 days for 4 cycles; calculate the dose of carboplatin using the Calvert formula |
| 4 | Pemetrexed 500 mg/m^2^ plus cisplatin 75 mg/m^2^, IV | Day 1 every 21 days for 4 cycles |

AUC, area under the curve; IV, intravenous
